# Understanding Long-term Outcome from the Patients’ Perspective: A Mixed Methods Naturalistic Study on Inpatient Psychotherapy

**DOI:** 10.5334/pb.432

**Published:** 2018-10-04

**Authors:** Melissa De Smet, Reitske Meganck

**Affiliations:** 1 Department of Psychoanalysis and Clinical Consulting, Ghent University, Ghent, BE; 2 Aspirant of the Flanders Research Foundation (FWO), BE

**Keywords:** psychotherapy outcome, patient perspective, mixed methods research, naturalistic research, inpatient psychotherapy

## Abstract

**Objective:**

The complex phenomenon of psychotherapy outcome requires further conceptual and methodological developments that facilitate clinically meaningful research findings. In this study, we rely on an idiosyncratic and process-oriented understanding of treatment effects in order to investigate long-term outcome. A conceptual model of long-term outcome is presented that comprises both a taxonomy of change and explanatory factors.

**Method:**

A mixed methods naturalistic study was conducted in an inpatient psychotherapy setting. Long-term quantitative outcome data are complemented with a data-driven thematic analysis of interviews with 22 participants, five to six years after ending inpatient psychotherapy.

**Results:**

Long-term outcome findings show improved well-being for the majority of former patients and this until five to six years after treatment. From the patients’ perspectives, long-term changes can be situated on different interrelated existential levels: reconnection to others and (the meaning of) life, a revelation, an altered self, life changes, and altered expectations and ideas about recovery and treatment. The complex interplay of the person, the therapy centre, the outside world and the evolution over time helped explain the experienced changes and individual differences.

**Conclusion:**

The findings support the value of an idiosyncratic and process-oriented understanding of outcome and recovery as well as substantiate the importance of multiple methods and perspectives when studying the effects of psychotherapy.

## Introduction

Understanding the effects or outcome of psychotherapy is essential for both research and clinical practice, yet the on-going discrepancy between both fields impedes a mutual dialogue (Castonguay, Barkham, Lutz & McAleavey, 2013; Kazdin, 2008). As outcome research has a huge impact on clinical practice through health care policy (APA, 2006; McLeod, 2016), the question of how to define and determine the effects of therapy remains a central issue. Evidence-Based Practice emphasises ‘the *integration* of the best available research with clinical expertise in the context of patient characteristics, culture, and preferences’ (APA, 2006, p. 273; emphasis added), yet in terms of policy this is often limited to the “best available evidence”, which in turn is predominantly reduced to highly standardized efficacy studies. Unsurprisingly, given the complexity of clinical practice in general and treatment outcome in particular, this tendency is heavily criticized in both the academic literature and the clinical field (Gaudiano & Miller, 2013; Hill & Lambert, 2004; Westen, Novotny & Thompson-Brenner, 2004). To say the least, further conceptual and methodological developments are required in order to curtail the divide between research and clinical practice (Kazdin, 2008; McLeod, 2011).

In studying the effects of therapy, outcome has traditionally been separated from the process of treatment (Hill & Lambert, 2004), resulting in an arbitrary differentiation. This misconception has ‘clouded the thinking regarding research design’, especially in outcome research, as ‘to some extent process research is outcome research, and outcome research is equivalent to process investigation’ (Kiesler, 1966, p. 126). A unidirectional relationship between process and outcome is furthermore questioned (Stiles & Shapiro, 1989): ‘process measurements taken from on-going therapy may be the result [outcome] rather than the cause [process] of clients’ improvement’ (Stiles, Shapiro & Harper, 1994, p. 42). Long-term follow-up studies have shown the value of investigating treatment outcome over a longer period of time, looking at treatment effects in a process-oriented manner, yet to date such long-term outcome studies remain limited (Ekroll & Rønnestad, 2016).

The impact and consequences of a demarcated or narrowed conception of outcome are most apparent in the so-called gold standard research design, the randomized controlled trial (RCT). High levels of standardization (e.g., strict in- and exclusion criteria, treatment protocols; Westen et al., 2004), an over-reliance on symptom-oriented outcome measures (Braakmann, 2015; Levitt, Stanley, Frankel & Raina, 2005), and the selection of one primary outcome measure to determine treatment outcome (De Los Reyes, Kundey & Wang, 2011), clearly simplify the understanding of treatment effects. Moreover, changes in outcome scores may not represent or correspond to an individual’s functioning in daily life, nor can it give insight into how an individual interpreted the questions asked. Standardized outcome measures are therefore considered “arbitrary metrics” requiring real-life contextualization (Blanton & Jaccard, 2006; Hill, Chui & Bauman, 2013; Kazdin, 2006).

Ergo, a growing interest in qualitative and mixed methods research emerges, as they allow for the contextualization of research findings and in-depth exploration of complex phenomena (Binder et al., 2016; Kazdin, 2008; Klein & Elliott, 2006; Midgley, Ansaldo & Target, 2014). In this regard, the perspective of the patient undergoing treatment is increasingly considered an essential source of information (Binder, Holgorsen & Nielsen, 2009; McLeod, 2016; Strupp & Hadley, 1977; Valkonen, Hanninen & Lindfors, 2011). This *first*-person perspective stands for the subjective experience, personal view or opinion of patients and must be considered distinct from a researcher or *third*-person perspective represented in the pre-defined questions and answers of standard outcome scales (Englebert, Follet & Valentiny, 2017; Englebert et al., 2018; Hill et al., 2013).

Opposed to standard outcome research, qualitative inquiry of patient perspectives has revealed important dimensions of change other than symptomatic changes (McLeod, 2011), for instance pointing at existential and interpersonal domains (cf. Binder et al., 2009; Nilsson, Svensson, Sandell & Clinton, 2007). Important individual differences in the experience of outcome have also been observed depending on patients’ personal background (Kühnlein, 1999; Valkonen et al., 2011) and the type of therapy (Nilsson et al., 2007). Moreover, it seems that the effects of psychotherapy must be understood in relation to the broader life span of patients (Midgley, Target & Smith, 2006) and other life domains (Binder et al., 2009). Together these studies show the relevance of investigating the patients’ perspective on psychotherapy and outcome, the importance of a broader conception of treatment effects, and the value of understanding outcome within an idiosyncratic context.

The current study wishes to contribute to these lines of research within the particular context of inpatient psychotherapy, a setting that challenges the field with an even higher level of complexity due to its particular characteristics. The different therapy groups in inpatient treatment can create multiple therapeutic climates (Tschuschke & Dies, 1994), and various elements outside the therapy sessions (e.g., living together on a ward) can facilitate change Leszcz, Yalom, & Norden (1985). Hence, inpatient care cannot be considered equivalent to the sum of its parts. Additionally, inpatient populations are typically more complex, as they often suffer from more chronic mental difficulties and/or are situated within more complex social contexts compared to patients in outpatient psychotherapy. Due to these specific characteristics, the setting does not lend itself well for controlled research and has mostly been investigated by means of basic pre-post measurements (Kösters, Burlingame, Nachtigall & Strauss, 2006). Consequently, little is known about the particular effects of inpatient treatment and change processes over time. Using multiple methods and inquiring about patients’ perspectives, can therefore offer a major contribution to the investigation of this specific and complex setting. In line with current ideas on practice-based evidence (Castonguay et al., 2013), we further believe naturalistic bottom-up inquiry offers important deepening and real-life knowledge, benefitting both research and clinical practice. Ultimately, practice-based research has the potential to circumvent the difficult translation of the context of research into the context of clinical practice; at the same time addressing the sometimes-pressing influence of health care policy (cf. managed care) on the organisation of health care (Gaudiano & Miller, 2013).

In the context of inpatient treatment, the current study aims at understanding long-term outcome within the broader, personal life context of patients. Following a process-oriented understanding of outcome, we investigate which changes former inpatients have experienced during and after inpatient psychotherapy (RQ1) and which factors (in or outside treatment) they believe have influenced these changes (RQ2). Finally, we examine how the perspective of former inpatients complements findings of long-term outcome based on quantitative measurement (RQ3). To answer these objectives, a mixed methods design is deployed, incorporating quantitative long-term outcome findings and qualitative inquiry of patients’ perspectives on changes over time. In an attempt to facilitate the implementation of findings into clinical practice, the present study takes place in a naturalistic setting.

## Methodology

### Setting

The study relies on naturalistic data gathered in an inpatient psychotherapeutic centre in Antwerp, Flanders (Belgium). The therapy centre offers intense, long-term inpatient psychotherapy to individuals with chronic mental difficulties, predominantly in the range of anxiety and mood disorders, and often in the context of attachment issues and personality disorders (cluster C). The overall approach of the treatment centre requires a certain level of stability, autonomy and self-reliance of patients in order to engage in the intense psychotherapeutic programme. For this reason, the centre must be considered distinct from a classical psychiatric ward; people suffering from acute psychological problems are referred.

After two initial intake interviews, a person engages in an exploratory treatment period of four weeks during which all forms of therapy are explored; only a minority is referred after this phase, when treatment is deemed to be ill-suited. During treatment, each patient receives an individualized programme consisting of four to five forms of therapy, provided in six to nine sessions of individual or group therapy per week. Therapy is based on different frameworks and provided by trained and specialized therapists in cognitive behavioural, systemic and psychoanalytic therapy. Additionally, creative therapy is provided in the form of dance, movement, drama, visual and music therapy. Regular meetings with a psychiatrist are held regarding drug treatment; a personal mentor is the patient’s contact throughout the treatment process. Finally, considerable free time is available for sports and other creative activities, all taking place in a quiet and natural surrounding environment.

### Participants

Between September 2009 and August 2010, all individuals entering the treatment centre were asked to participate in an internal outcome study. At consecutive time points (i.e., at intake, every two months of treatment, post treatment, one-year and five-year follow-up) outcome was evaluated using the Outcome Questionnaire-45 (OQ-45, Lambert et al., 1996; de Jong et al., 2007). Forty-three participants entered the study; nine dropped out of the study (i.e., no post and follow-up data available). The remaining group (*n* = 34) provided data with variable response rate: 27 respondents at the end of treatment and 29 at one-year follow-up. The qualitative strand of this study focuses on the second follow-up inquiry, conducted five to six years after treatment termination. For this study, 27 participants (with nearly complete data) were contacted, 24 responded to our call for participation and 22 were willing to take part in the follow-up interview.

The research sample consisted of 19 women and 3 men, ranging in age from 31–61 (*M* = 47.95; *SD* = 8.94); all were of Caucasian European decent. Nine participants completed secondary education, 12 had degrees in higher education; seven of the participants were married, three were living together with their partners, and 11 participants indicated they were single (of which six were divorced) at the time of the interview (one participant did not mention her marital status). Eleven of the participants were not working at the time, five of them were retired (often due to medical reasons), were registered as disabled, and one was unemployed at that time. The rest of the participants were employed, of which one was self-employed; three did voluntary work.

Patients were diagnosed at the start of treatment according to DSM-IV-TR (American Psychiatric Association, 2000). The recorded Axis I and Axis II diagnoses were: depressive disorder (*n* = 14), somatization disorder (*n* = 5), relational problems (*n* = 4), anxiety disorder (*n* = 1), PTSD (*n* = 2), adjustment disorder (*n* = 5), personality disorder NOS (*n* = 12), dependent PD (*n* = 4), avoidant PD (*n* = 4), obsessive compulsive PD (*n* = 3) and borderline PD (*n* = 1). The majority (*n* = 18) stayed for the maximum period of 12 months in treatment, one person ended treatment after six months, two people after nine months and one person stayed for a period of ten months. Table 1 gives a summary of the research sample’s treatment before and after inpatient treatment (based on patients’ self-report). Appendix 1 offers a more detailed idiosyncratic description of the participants (in line with Thurin & Thurin, 2007), comprising a brief clinical description, diagnoses and the change in outcome scores per patient.

**Table 1:** Treatment history of research sample.

Treatment history	Before inpatient treatment	After inpatient treatment	At five-year follow-up

Participants	n	n	n

Outpatient psychotherapy	17	13	8
Psychiatrist	18	9	10
Medication	16	6	7
Psychiatric hospitalization	7	4	

*Note. n* = 22; various treatments per patient. Outpatient psychotherapy: often long-term, many different therapists and forms of talking-therapy or alternative treatments were consulted before inpatient treatment; creative therapy was increasingly consulted after inpatient treatment. Medication (in order of occurrence): antidepressants, sleep medication, anxiolytic, antipsychotic. Psychiatrist: if not specified, psychiatrist provided counselling.

Both the ethical committee of health institution Emmaus and the Ethical Committee of the University Hospital of Ghent University (Belgium) (registration number: B670201524637) approved the project. Every participant signed a written informed consent, for both the initial quantitative study and the follow-up inquiry.

### Instruments

Before the follow-up interview, participants completed a form asking for information about gender, age, occupation, medication use and psychotherapeutic treatment (before and after treatment).

**The Outcome Questionnaire (OQ-45; Lambert et al., 1996; de Jong et al., 2007).** The OQ-45 is one of the most frequently used self-report instruments for measuring patient clinical distress and well-being. The measure consists of three dimensions: subjective (symptomatic) distress (intra-psychic functioning), interpersonal relations and social role. Forty-five items are filled out on a 5-point Likert scale (0 = not at all; 4 = always). Sub-scores are calculated for the three sub-categories, the sum of which gives a total-score, ranging from 0 to 180; higher scores reflect greater distress. The Dutch version of the OQ-45 shows good internal consistency (a = 0.96; total score), good test-retest reliability after two to three weeks (*r* = 0.82; total score for patient population) and sufficient concurrent validity (de Jong et al., 2007).

**Semi-structured interview.** The first author administered an adjusted version of the semi-structured **Client Change Interview** (CCI; Elliott, Slatick & Urman, 2001). The interview guide was constructed to evoke participants’ experiences of the period before the inpatient treatment and the reasons for consulting the therapy centre, the changes they believe occurred during and after this treatment period, and what they believe influenced these changes. Interviews were audiotaped and transcripts were analysed.

### Procedure

An explanatory sequential mixed-method study (Hesse-Biber, 2010) was conducted. The quantitative outcome scores and the narratives of former inpatients were analysed separately and integrated at the phase of interpretation, allowing for a complementation and deeper understanding of both the quantitative and qualitative outcome findings. This design can be summarised as “quan → QUAL”: a qualitative strand was added secondary to an existing quantitative study yet becoming the most important focus of the explanatory study (cf. Hesse-Biber, 2010).

The majority of the interviews were conducted at people’s homes, a few were held in the therapy centre and one was administered at the faculty of Psychology and Educational sciences (Ghent University). Interviews lasted 100 minutes on average. An initial in-depth analysis was conducted on a sub-selection of 13 rich cases (displaying diversity in experiences), followed by an analysis of the remaining interviews (*n* = 11) using the initial themes as a compass. Minor adjustments were needed in order to encompass all narratives in a single final model of long-term outcome.

### Long-term quantitative outcome classification

Participants were classified in terms of reliable change and clinical significance based on four categories: clinical significant change (CS), reliable change (RC), no RC and deterioration (Jacobson & Truax, 1991). The outcome scores of the inpatient population were compared to Dutch norms (de Jong, Nugter, Lambert & Burlingame, 2008). In order to reach reliable change for the OQ-45 total score, a person must show a decrease in scores equal to or larger than 14. The cut-off between the clinical and nonclinical population for the Dutch OQ-45 is set at 55 (based on the test-retest reliability of 0.96; de Jong et al., 2007). We present all available quantitative outcome data (i.e., starting from the entire sample participating in the initial study) measured at the start of treatment (*n* = 43), at treatment ending (*n* = 27), at one-year (*n* = 29) and five-year follow-up (*n* = 22). To control for potential effects of drop-out, we compared the averages score on the OQ-45 (at the start of treatment) in the drop-out group (*n* = 9) and the remaining group (*n* = 34) by means of an independent samples t-test. Dependent samples t-tests were used to verify whether the outcome scores for the remaining group differed statistically significant at the end of treatment, one-year follow up and five-year follow up compared to the start of treatment.

### Qualitative analysis

A data-driven Thematic Analysis (Boyatzis, 1998; Braun & Clarke, 2006) based on principles of Grounded Theory (Glaser & Strauss, 1967), was conducted. This form of inquiry enabled the exploration of the phenomenon of long-term outcome in the participants’ terminology and to identify themes in the data in a bottom-up manner. In our understanding of the first-person perspective, we adhere to a contextual idea: people give meaning to their experiences yet are influenced by the broader social context (Boyatzis, 1998; Braun & Clarke, 2006). The analysis focused on the macro-process of psychotherapy, i.e., a wide angle in contrast to the analyses of micro-processes (e.g., specific therapy effects). Moreover, the study aimed at investigating the subjective experience of several different participants (i.e., between-case variation) (Denzin & Lincoln, 2005).

### Phases of analysis

Both authors coded the interviews separately; after each phase of analysis discussions were held until agreement was reached through a consensual process (cf. Hill, Thompson & Williams, 1997). The process of inquiry contained the following steps that were moved along in an iterative manner:

1. Getting familiar with the narratives: emerging research question
  While conducting and reading the interviews, important individual differences among former inpatients’ experiences became apparent. Subsequently, this variety among participants was incorporated in the analysis of experienced changes and RQ2 was further specified: *how can the explanatory factors help to understand individual differences between former patients’ experienced changes?*
2. Identifying relevant meaning units (initial sample, n = 13)
  Meaning units were identified (cf. Giorgi & Giorgi, 2003) by selecting chunks of text entailing all forms of experienced change (RQ1) and all mentioned influential factors in or outside treatment (RQ2). In this phase, we attempted to keep our perspective as broad as possible to prevent excluding relevant information too soon. Non-relevant parts, i.e., not dealing with subjects like well-being, experienced change or the therapy centre, were omitted.
3. Providing codes for the selected meaning units (initial sample, n = 13)
  We attached keywords to text segments (Kvale & Brinkmann, 2009), systematically narrowing down the number of codes by integrating and renaming codes to the final version. For example, “being less insecure” and “more confidence” were merged together in a following step, resulting in the final category of “empowerment” (Mortelmans, 2011). RQ1 and RQ2 were coded for simultaneously, yet by using different indications or colours they were visually and thematically separated.
4. Clustering codes to build core and subcategories (initial sample, n = 13)
  The clustering of themes and factors was based on a thematic understanding of the perspective of all 13 participants and was conducted in several phases, in order to find the best-suited representations (e.g., the term “mollification” was chosen over “softening” or “appeasing” based on its definition “to soothe in temper or disposition”; Merriam-Webster online dictionary). At this phase, the first author reread the 13 interviews in order to verify whether the themes and factors still resonated with the participants’ narratives.
5. Preliminary taxonomy and explanatory model (initial sample, n = 13)
  Two separate preliminary models resulted: a taxonomy of experienced change (RQ1) and an explanatory model (RQ2). Both were developed to represent an overall representation as well as patients’ individual differences. This resulted in twofold subcategories (e.g., “an altered self” consists of “empowerment” and “mollification”).
6. Coding the second part of the interviews using the initial categories as a compass
  Minor differences were found between the preliminary model and additional interviews and adjusted by adding subcategories (for example “A new perspective”) and renaming categories so terms would fit the uttered meanings of the entire sample.
7. Resulting conceptual model of long-term outcome: merging models
  Ultimately, we agreed on merging both the taxonomy of change and explanatory factors into one overall model, as this allowed a clearer visual representation of the interrelations and influences as experienced by the participants. After this final phase, relevant citations were selected to provide rich examples.

### Reliability and validity

In function of the reliability of the study, we tried to remain transparent about the entire process (Stiles, 1993) and acknowledge the influence of the perspective and background of the researchers. The first author conducted the interviews, making her personally involved with the participants and consequently also with the therapy centre. By avoiding preparatory knowledge (on the treatment setting and the quantitative outcome results), the influence of this personal involvement was countered as much as possible. The researchers’ personal interest in idiosyncratic experiences clearly navigated the focus of the study and analysis. By making this explicit, consequences of implicit guiding assumptions were controlled as much as possible (Creswell & Miller, 2000). In function of the validity of the study we worked in a systematic manner to form conclusions and interpretations (Stiles, 1993). Triangulation among researchers, several interviews (divided into two subsamples), and quantitative and qualitative indications of outcome were applied to gain different perspectives on the issue. The ultimate themes were formed by asking critical questions regarding codes and categories (Mortelmans, 2011). We attempted to stay open for any information coming from the narratives throughout the entire process (cf. non-framing interview format).

## Results

We first present the quantitative long-term outcome data from the start of therapy to five-year follow up and look into patients’ perspectives on change and explanatory factors thereafter. Interpretations of the separate findings will be described in the result section; broader integrative conclusions and implications will be presented in the discussion.

### Long-term quantitative outcome

Table 2 summarizes the change in average total scores on the OQ-45 over time, the standard deviations (SD) and range in scores (i.e., minimum and maximum score) as well as their meaning in comparison to a clinical and normal population. Table 3 shows the number of patients in the clinical and functional range, as well as CS change, RC, no change and deterioration.

**Table 2:** The average, variation and meaning of outcome scores measured with the OQ-45 (total scores).

Average total score (OQ-45)	Research sample		Clinical population		Normal population	

	M	SD (range)	Meaning	Percentile	Meaning	Percentile

**Start therapy**	97	16.5 (60–144)	High	80–95	Very high	95–100
**End therapy**	67	22.2 (36–115)	Below average	20–40	High	80–95
**One-year FU**	73	22.7 (24–124)	Average (men) Below average (women)	40–60 20–40	Very high	95–100
**Five-year FU**	76.7	19.6 (30–116)	Average	40–60	Very high	95–100

*Note*. Research sample: *n* varies across measuring points. Clinical population: 628 men and 896 women; Normal population: 296 men and 511 women (de Jong et al., 2008).

**Table 3:** Clinical significance of outcome scores measured with the OQ-45 (total scores).

OQ-45 total score	Start therapy	End therapy	One-year FU	Five-year FU

	n	n	n	n

Participants	43	27	29	22
Functional	0	9	6	3
Clinical range	43	18	23	19
Change from start treatment				
CS		9	6	3
RC		12	15	11
No RC		5	5	6
Deterioration		1	3	2

*Note. n* varies due to the varying response rate at each measurement. Functional distribution: below clinical cut-off; clinical range: above the clinical cut-off; CS: reliable change and crossing the clinical cut-off; RC: reliable change only; No RC: criteria of statistical reliable change not met; Deterioration: reliable deterioration (reliable change in negative direction). The cut-off between clinical and non-clinical population for the OQ-45 = 55; reliable change on the OQ-45: difference in scores ≥ 14.

The average total outcome score decreased from start to end of treatment, and slightly increased again during the follow-up period. The decrease in scores showed to be statistically significant at all time points (for *n* = 34) with an average decrease of 30 points (95% CI: 21.01–39.1; p < 0.01) from start to end of treatment, 23.2 points (95% CI: 14.38–32.1; p < 0.01) from start to one-year follow up and 18.7 points (95% CI: 7.68–29.69; p < 0.01) from start to five-year follow up. The average distress level in the drop-out group (*M* = 100.56; *SD* = 12.053) and remaining group (*M* = 96.68; *SD* = 17.728) did not differ significantly (*M* = –3.879; *SD* = 6.287; 95% CI: –16.577–8.818, p = 0.274); the decrease in scores thus does not seem influenced by the participants who dropped out from the study. Despite the significant diminution in severity of distress over time, the average score remains high in comparison to a normal population. In terms of clinical significance, the changes in scores indicate the majority of participants have improved reliably, and this until five-year follow-up, yet only a minority reaches and remains the level of clinical significant change. The large range in scores further suggests a strong variety in the level of functioning among participants at all measurement points.

### Conceptual model of long-term outcome of inpatient psychotherapy

**Figure 1:**
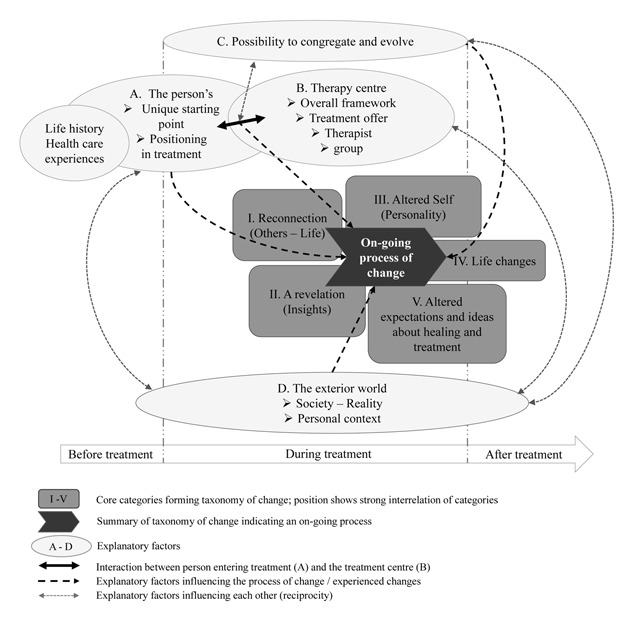
Conceptual model of long-term outcome comprising the taxonomy of experienced changes (I–V) and explanatory factors (A–D).

Figure 1 presents the model of long-term outcome that comprises both a taxonomy of change and explanatory factors. In order to indicate variation in the prevalence of the categories and factors we rely on different phrasings: “(nearly) all” participants, indicates 90–100%, “most of” or “the majority” stands for 80–90%, “many” or “often” indicates 60–80%, “some(times)” or “others” means about 30–50%, and “a few” ranges around 10–20% of the participants.

#### Taxonomy of change: an on-going and non-linear process of existential changes

Table 4 presents the taxonomy of the experienced changes during and after inpatient treatment, differentiated in five categories that represent different levels of existential change: I. Reconnection to others and (the meaning of) life, II. A revelation, III. An altered self, IV. Life changes, and V. Altered expectations and ideas about recovery and treatment. In the conceptual model of long-term outcome presented in Figure 1, these changes are depicted along a timeline, indicating changes occurred at different moments in time. Without making causality claims, former patients described how the initial changes (i.e., I & II) have helped them engage in treatment and facilitated later changes (i.e., III, IV & V). In the resulting model, the experienced changes are positioned closely to each other, as they must be understood as strongly interrelated, i.e. changes coincided and influenced each another.

**Table 4:** Taxonomy of the experienced changes.

Core and subcategories

*I. Reconnection to others and (the meaning of) life* i. A feeling of belonging ii. A new perspective *II. A revelation* i. Insight into self and difficulties ii. Alternative ways of expressing emotions and thoughts *III. An altered self* i. A mollified self ii. An empowered self *IV. Life changes* i. Concrete changes in dealing with or handling things in life ii. Life-altering changes *V. Altered expectations and ideas about recovery and treatment* i. Recovery as on-going and fluctuating process ii. Resignation versus disappointment

##### I. Reconnection to others and (the meaning of) life

Foremost, nearly all former patients experienced ***a feeling of belonging*** at the inpatient treatment centre. They described how they could recognise themselves and their difficulties in fellow patients, were accepted for who they are and felt worthy. Furthermore, patients gained a new perspective on life, were filled with renewed energy and enthusiasm for life, and gained hope and courage to go on and overcome difficulties. Together these changes were interpreted as an experience of ***reconnection***, which is in stark contrast to the *disconnection*, i.e., exclusion, loneliness, failure and hopelessness, people experienced in society before entering inpatient treatment. For many, this feeling of reconnection was not only an important condition for engaging in therapy, but also meant a lasting change as such.

‘What I remember very well is the aspect of being heard, being respected and treated as normal. I always had the feeling there was something wrong with me, that I was not okay, or it was not good enough, I had to try harder or they did not want me; I did not belong. At [the inpatient therapy centre] that all vanished. Now I feel at home anywhere I go.’

##### II. A revelation

Secondly, for nearly all participants, the treatment period lead to ***a revelation***. First of all, people mentioned “eye-openers” or ***insights regarding themselves and their difficulties*** as one of the most important changes.

‘The biggest change for me was that, you think you know what your problem is, but you notice it can be something completely different. (…) At a certain point, you notice it goes much deeper.’

Also, people described how the various creative forms of therapy offered them ***alternative ways***, or even a first opportunity, to express emotions and thoughts; many continued to use these new ways after the treatment period (in the form of therapy, taking specific courses or hobbies).

‘To the outside world I have always been the “ever-smiling person”, I did not know anger and swallowed my frustration. Here [in the inpatient therapy centre] it changed a lot. I have learned to give words to my feelings, by first painting them on a canvas or by making something in clay.’

##### III. An altered self

At a later stage in treatment and to a lesser extent after treatment, patients experienced changes in their personality. On the one hand, many participants had become milder for themselves and others, showing a process of ***mollification***. Patients stated they have learned to acknowledge their own boundaries and accept difficulties that happened in the past, by taking them seriously.

‘I have become softer, or that’s what I am being told now by others. At my job, I now indicate my limits beforehand, as if I *allow* myself a private life now. (…) And I have learned it’s okay to do ‘nothing’ for a while.’

On the other hand, many of the participants indicated they have become more independent, stronger and gained self-esteem, reflecting a feeling of ***empowerment***.

‘I have learned to stand up for myself, to choose for myself. I have been able to take more distance from my parents, with the feeling: “it’s okay; it’s okay to think about yourself, to take yourself seriously”.’

##### IV. Life changes

Experienced changes at the level of personality coincided with important ***concrete changes in dealing with or handling things in life***, increasingly taking place during and after treatment.^1^

‘Since then [the inpatient treatment] I have always gone home [from work] on time and never took my computer with me. At five o’clock I could say: “it’s time to go home, I’m out.” Before, coming home meant turning on the computer and I could keep on working.’

Apart from changes in employment, also housing was often reconsidered and changes in family constitution and intimate relations (e.g., new partner or divorce) occurred. For some of the participants these changes were ***life-altering***, as the period of inpatient psychotherapy truly meant a turning point in life.

‘My entire world has changed by coming here [the inpatient therapy centre]. I opened up here, my social life started and subsequently I met my husband. After years of invalidity and depending on my parents, my husband and I now live together and have a little toddler running around. I still feel tired, but I have people that rely on me now.’

##### V. Altered expectations and ideas about recovery and treatment

Nearly all of the participants disclosed they have been dealing with severe difficulties throughout their lives and none of them stated they were “cured”. However, during and after the treatment period a ***change in ideas and expectations about recovery and treatment*** occurred. The initial idea that after a couple of months all problems would be solved, changed for most patients to perceiving recovery as ***an on-going and fluctuating process***. Apart from the many valuable changes, and independent from the situation at the time of the interview, many participants also experienced a breakdown or recurrent difficulties.

For most of the participants, seeing recovery as an on-going and rather fluctuating process functioned as a ***resignation***, leading to adjusted (i.e., more realistic) expectations and understanding of their own process of recovery, subsequently influencing concrete changes in life and coinciding with changes in personality (III & IV). A few participants, however, seemed to hold on to a more fixed idea about recovery or had an ideal image of treatment, eventually resulting in a ***disappointment***, in themselves or in psychotherapy, as these (sometimes high) expectations were not met.

‘I believed all my problems would be solved: I would easily find work and build new social contacts, I would have a better relationship with my parents. But I expected too much. My image about the world and future had become too idealistic.’

#### Explanatory factors of change: understanding individual differences

Figure 1 depicts four broad factors in patients’ narratives that have explanatory value with respect to the experienced changes and their interrelation: A. The person’s unique starting point and positioning in treatment, B. Central elements of the therapy centre (in relation to the person), C. The possibility to congregate and evolve, and D. The relation to the exterior world. These factors must be understood as evolving throughout and after the treatment period and are thus also depicted along the timeline in Figure 1.

##### A. The person’s unique starting point and positioning in treatment

Important differences were observed among former patients regarding *when or where* in the course of their lives and health care trajectory they had entered inpatient treatment, making up ***the person’s unique starting point***. Whether the inpatient treatment followed previous psychotherapeutic experiences or not, consequently influenced the process of inpatient therapy, the extent to which insights were gained and changes in personality and life could occur.

‘At that moment [in inpatient treatment] I was really ready for it, and I really wanted to do something about it. A bit opposed to the years before, I think. When I was hospitalized in psychiatry they mainly had to keep me alive. This [the inpatient therapy centre], however, has been my final hospitalization.’

Secondly, former patients described different ways of ***positioning oneself in treatment*** that could be summarized as: dependent-passive, assertive-active, overactive, and independent – keeping a distance. These four positions are neither mutually exclusive nor fixed yet present a predominant way of engaging in treatment; Table 5 provides examples of every position. Whether someone displayed a high need of guidance, engaged actively in treatment, was preoccupied with gaining instant effects, or had difficulties opening up in treatment (e.g., due to trust issues), influenced the treatment process differently and subsequently also the experienced changes.

**Table 5:** Examples of different positions in treatment.

Position in treatment	Example excerpt

***Dependent-passive:*** *high need of guidance, support and initiative from therapists*	‘It was hard when the initiative had to come from me. Maybe it’s my personality; I often need a lead, as I often tend to lean on others. And maybe it was the plan of the therapists that I would take initiative and stand up for myself. But having to create things myself in therapy was hard.’
***Assertive-active:*** *highly engaged in treatment process, taking own initiative*	‘Being here [in the inpatient therapy centre] was a unique opportunity I had to grasp with my both hands. I chose for myself, I wanted to become a happier person, so I engaged in everything they offered me. At a certain point, I even asked for an extra form of therapy.’
***Over-active:*** *preoccupied with “working hard” and time-efficiency of treatment*	‘I had prepared myself for [the inpatient] treatment. I had made drawings with all my characteristics on it, I wanted to know all of it, I did not want to do nothing there [in the inpatient therapy centre]; I wanted to work on myself.’ ‘When I was drawing in therapy I thought: “I have to be at my department, I have to lead my team, my colleagues!”’
***Independent-keeping a distance:*** *avoiding participation or inhibited to fully engage*	‘I’ve realized, I’m not very good at therapy… Personal conversations with people I don’t know so well are hard, it takes a while before I trust a person. I’m very secretive. I constantly censor myself and consider ‘what can I share and what not’ and “shouldn’t I be solving this on my own”.’

##### B. Central elements of the therapy centre (in relation to the person)

A reciprocal arrow between the person and therapy centre in Figure 1 signifies the importance of the interaction between both factors. Specifically regarding the interaction of the patient and the ***overall approach*** of the therapy centre, people who described a more *dependent-passive* and *independent-keeping a distance* position stated to have encountered the most difficulties in displaying autonomy and self-reliance. Patients with an *assertive-active* position seemed to engage more easily in a process of self-exploration, while people in an *over-active* position encountered difficulties with the dimension of taking time for oneself (e.g., due to a feeling of not working hard enough); see Table 5.

Accordingly, the extent to which the treatment was experienced as well adjusted to personal needs differed from participant to participant, yet all mentioned at least one therapy in their ***treatment package*** they had found helpful (cf. adjusted therapy programme). The connection with the ***therapist*** (and by extension the personal mentor and psychiatrist) and the ***group*** appeared crucial for changes to occur.

‘It was hard for me to open up, and most of them [the therapists] let me be, but there was one therapist who saw through me. He said: “you are here long enough, you can make it a little bit harder on yourself”, and I thought “he is right”. The fact that he did not tolerate my attempts to hide [was important]’

##### C. The possibility to congregate and evolve

The person, the treatment and their interaction must not be understood as fixed. The possibility to evolve towards a coming together of both (i.e., harmony and accordance) appeared essential to understand why changes came about or not. For instance, many described how they have evolved in the position of being in treatment (e.g., from passive to active; overcoming uncertainty and distrust), or how therapy was altered to reach a patient’s (changing) needs (i.e., via therapists’ interventions or by changing a therapy programme). A rift in the relation or evolution, on the other hand, could lead to the undermining of further treatment and change.

‘After nine months, they [the staff] told me: “we cannot continue to work with you, we are stuck.” And I knew it myself. They wanted me to participate actively but my reaction was to retreat, to build a wall around me. And they were right, no one could get in.’

##### D. The relation to the exterior world

Participants described their experiences explicitly in comparison and in relation to ***society*** or ***reality*** outside the therapy centre. To many, the period in the inpatient treatment centre offered a healing experience and buffer against hostile encounters (e.g., discrimination, stigmatization, high demands and pressure), although some also suffered from this contrast with the outside world.^2^

‘I miss the warmth of the therapy centre; people listened to you, it was okay to say “I’m not feeling very well today”. In society, this is just not tolerated, if you state you’re not doing okay you are pushed aside; you always need to be strong.’

Furthermore, the ***personal context*** (i.e., family, social and professional context) had an important influence on the treatment and change process. For some, this context was a reason for being hospitalized and distance as such was salutary; to others, the context was left aside too much during the treatment period. The extent to which, for instance, family, social life and further career planning had been integrated into the treatment period seemed an important facilitator of change and transition to daily life after ending treatment. For some patients, this was however beyond their possibilities at this point in their treatment trajectory (cf. unique starting point; A). Further, family at home sometimes meant an important reason to prematurely end therapy.

‘My daughter asked me to come home again, as I had promised my treatment would maximally take one year. But actually, my treatment wasn’t entirely finished then. Now I regret that [premature treatment termination].’

## Discussion

Long-term outcome findings show improved well-being for the majority of former patients and this until five to six years after ending inpatient treatment. Yet, in order to obtain a deeper understanding and contextualization of these findings, long-term outcome scores were complemented by the patients’ own perspective.

The conceptual model based on the qualitative analysis of patients’ narratives indicates long-term outcome can be understood as an ongoing and fluctuating process of predominantly existential changes. These changes are furthermore strongly interrelated and were described as coinciding and influencing each other over time (cf. Binder et al., 2009; Ekroll & Rønnestad, 2016). In line with previous (mostly outpatient) qualitative research, participants in this study mentioned changes in the domains of the self (in terms of personality), interpersonal relations (here more broadly described as the exterior world), and specific life conditions (Klein & Elliott, 2006; Midgley et al., 2006; Nilsson et al., 2007). Concrete life changes seem to correspond to behavioural changes mentioned in other studies, while alternative ways of expressing emotions and thoughts may be seen as different ways of coping (Ekroll & Rønnestad, 2016; Midgley et al., 2006; Nilsson et al., 2007). Accordingly, we observed a decrease in treatment intensity (i.e., medication, counselling, and hospitalization) following the inpatient treatment, while creative forms of therapy and other activities increased. Central to our findings is that none of the participants considered themselves as “cured” during or after treatment, yet nearly all emphasized the importance of the inpatient treatment for obtaining fundamental changes. This seems to correspond to the observation that only a minority of patients reached clinically significant change in outcome scores, although the majority showed reliable improvement compared to their initial level of functioning. Interestingly, participants in this study evaluated their process of recovery in light of their own personal life story while rejecting the normative standards of society. The inpatient treatment centre offered a healthier and healing experience that was strongly contrasted with the outside world. Whilst previous qualitative studies found changes in terms of accepting own limits and weaknesses (Kühnlein, 2011; Nilsson et al., 2007), unique for this study seems to be patients’ different perspective on recovery and treatment.

This finding touches upon the ethical question of how recovery, functioning and dysfunctioning are conceptualized. In accordance with the altered perspective of the participants in this study, a broader view on recovery can be noticed in the organisation of (mental) health care: a dichotomous understanding of “health” versus “illness” is increasingly replaced by a definition of “positive health” (Delespaul et al., 2016; Huber et al., 2011; Slade & Longden, 2015). The latter emphasizes the possibilities and abilities, *given* psychological, physical and social challenges and is considered more in line with clinical practice (Delespaul et al., 2016). For most of the participants, this different appraisal of recovery offered resignation, while fixated ideals were met with disappointment. Conceptualizations of dysfunctioning and recovery also provide the (often implicit) assumptions underlying the measurements in psychotherapy and outcome research (Imel & Wampold, 2008; Rieken & Gelo, 2015; Slife, 2004). From a positive perspective on recovery, the individual process and evaluation becomes central and comparison to others is only possible to a certain extent (Slade & Longden, 2015). The research field faces the challenge of adapting to the shifting perspective on outcome and the growing body of idiosyncratic research (quantitative and qualitative) is clearly promising in that respect. The current study aimed at contributing to these developments in the context of inpatient psychotherapy.

### Implications for further research on outcome

Our findings highlight the multiplicity of long-term outcome, substantiating the relevance of a broader conception and measurement of treatment effects. The complex interplay of the patient, treatment, external context and evolution over time further indicates that experienced changes cannot be explained by single or fixed factors. In this respect, our results support a dynamic understanding of the patient-therapist relationship as suggested by Clarkin and Levy (2004), although “the therapist” requires a broader interpretation in our study (i.e., elements of the treatment centre). Despite its residential nature, the influence of the context outside therapy must also be considered in this dynamic patient-treatment relationship (cf. McLeod, 2016). Regarding the patient, our study points at the importance of a patient’s starting point in treatment (influenced by life experiences and previous health care), which relates to the concept of patient characteristics that is broadly considered the single most important factor for explaining treatment effects (for an overview, see Bohart & Wade, 2013). While the patients’ perspective is often explored after treatment, it is arguable that a more in-depth understanding of the patient’s starting position can enhance knowledge on tailoring psychotherapy to individual needs (Norcross & Wampold, 2011). Qualitative analysis of patient’s experience before treatment could mean a valuable contribution here. Further, whereas most research assesses patients’ expectations statically, our study shows the potential relevance of evaluating treatment expectations as changing over time (Greenberg, Constantino & Bruce, 2006). Longitudinal prospective research would enable to look at individual trajectories of change in inpatient treatment (Bohart & Wade, 2013). The different positions in treatment that were found in this study (i.e., dependent, assertive, over-active and avoidant), seem to relate to several characteristics described in psychotherapy literature, like a patient’s “state of readiness”, level of engagement and possibility to establish good therapeutic alliance (Bohart & Wade, 2013). Further research could focus on how patients’ positioning in treatment evolves and can be facilitated during inpatient care (cf. individualized treatment programmes). The extent to which these different positions can be observed elsewhere requires replication in other settings. Likewise, our findings suggest a feeling of reconnection might be essential for patients to engage in treatment (cf. Ekroll & Rønnestad, 2016). The question can be raised whether this feeling is unique for this setting, specific for inpatient long-term treatment, or can also be observed in outpatient care and across different groups of patients.

### Strengths, limitations and future directions

The longitudinal design of this study enabled to outline the sustainability of changes over time; the idiosyncratic perspective of former inpatients allowed understanding these changes beyond symptomatic evolutions. Both can be considered important strengths of this study. High levels of complexity and diversity characterize the field of inpatient psychotherapy, and likewise, the treatment centre in this study cannot be considered identical to other inpatient settings (cf. Kösters et al., 2006). Nonetheless, several elements of residential care (e.g., living together on a ward; different forms of group and creative therapy) can be considered shared among inpatient facilities and therefore support transferability to a certain extent (e.g., Leszcz et al., 1985). Still, further research on inpatient settings is warranted to deepen the understanding of this particular therapy context. In the present study, we aimed to contribute to this inquiry by incorporating all available outcome scores and all 22 interviews, resulting in an overall-experience and general model of inpatient treatment. Investigating patients’ experiences of more specific aspects of inpatient treatment (e.g., the role of creative or group therapy), during or shortly after ending treatment, could complement our findings.

The participants in this study resemble a homogenous and local (Caucasian, Flemish) group of former patients. Studies that focus on specific (e.g., cultural, ethnic) or more diverse groups of patients (e.g., gender) could therefore offer an important contribution. Our participants also formed a sub-group of patients that were willing to participate in the study. This could imply that more dissatisfied people were not heard and that therefore relevant knowledge remains unexposed. Although we have incorporated negative experiences in our analyses (cf. Midgley et al., 2006; Nilsson et al., 2007), qualitative research focusing on negative outcomes remains scarce whereas it could offer an important contribution to the understanding of (inpatient) psychotherapy (e.g., Werbart, von Below, Brun & Gunnarsdottir, 2015). In line with this, effects of social desirable answers cannot be fully excluded (Thurin & Thurin, 2007).

Many external influences could have shaped former patients’ perspectives in the period of five to six years after ending treatment. Due to its naturalistic character, our study is limited in terms of standardization and control. For instance, the research sample is characterized by diverse clinical problems (e.g., diagnoses) unlike what is typical in standard outcome studies (Westen et al., 2004). The model of long-term outcome must thus be considered preliminary and we wish not to make strong causal claims regarding the effectiveness of treatment. Rather, the strength of a naturalistic research design is the clinically representative study of a particular context and population of patients, resulting into clinically valuable knowledge (Leichsenring, 2004). Finally, following Strupp and Hadley (1977), we agree that the chosen first-person perspective is only one perspective on outcome (e.g., compared to the therapist or societal perspective), and likewise only sheds light on certain aspects, while leaving others untouched. Nevertheless, studying the patients’ perspective offers major contributions to the clinical meaningfulness of research findings and understanding of psychotherapy outcome.

## Conclusion

This study offers support to the idea that, instead of relying on a unified and delineated definition and measurement of outcome or recovery, research and practice can greatly benefit from acknowledging the inherent complexity, multiplicity and diversity of psychotherapy outcome that require the incorporation of multiple methods and perspectives.

## Additional File

The additional file for this article can be found as follows:

- pb-58-1-432-s1.pdf
  **Appendix 1** Clinical description, diagnoses and total scores (OQ-45) per patient. DOI: https://doi.org/10.5334/pb.432.s1

## Footnotes

Note that the difference between “an altered self” and “life changes” is specifically situated on the level of the experience and feeling of “being different” (e.g., different mind-set, attitude and character) versus the concrete changes or actions a person undertook resulting from or coinciding with changes in personality (cf. categories are strongly interrelated).

The term “reality” was taken from patients’ own description. A distinction between the “outside world” (broadly termed as “the society”) and the “inside world” of the treatment centre was strongly made. This reality or outside world was mostly seen as “cold” and in stark contrast with the safe and caring environment of the treatment centre.
