# Supplementary material for: Understanding Long-term Outcome from the Patients’ Perspective: A Mixed Methods Naturalistic Study on Inpatient Psychotherapy
Source: Psychol Belg. 2018 Oct 4;58(1):276–96. doi: 10.5334/pb.432 (PMC6196577; doi:10.5334/pb.432)
Supplement: Supplementary file 1 [file pb-58-1-432-s1.pdf]

Appendix 1. Clinical description, diagnoses and total scores (OQ-45) per patient.

Demographics	Brief clinical description	Scores	
**Female, 45-50**	**Reason for consulting therapy centre:**<br>Severe relational problems (described as psychological terror, domestic abuse by ex-husband) leading to isolation and eventually a suicide attempt. Entered inpatient therapy centre after two months in psychiatry.<br>**Main difficulties:**<br>Highly depending of others, clinging (describes herself as "a geisha"), e.g., initial idea behind treatment was becoming the ideal wife for her ex-husband. Highly insecure, low self-esteem. Suffering from a lack of energy, negative thoughts, struggling with femininity.<br>**Diagnoses (DSM-IV):**<br>Depressive disorder; PD NOS	**Start**<br>**Post**<br>**1yFU**<br>**5yFU**	98<br>87<br>64<br>77
**Female, 50-55**	**Reason for consulting therapy centre:**<br>Severe depression. Several losses (close relatives) preceding the depressive episode together with difficulties at work, leading to what she calls "the ultimate crash". Entered treatment after three months in psychiatry.<br>**Main difficulties:**<br>Life history of physical and verbal aggression (in family and relationships); single mother after difficult marriage. Central: always taken care of others (at home, at work, in intimate relationships).<br>**Diagnoses (DSM-IV):**<br>Depressive disorder; Dependant PD; Avoidant PD	**Start**<br>**Post**<br>**1yFU**<br>**5yFU**	81<br>15<br>24<br>30
**Male, 45-50**	**Reason for consulting therapy centre:**<br>"Heavy crash" following difficult period: losing job, father deceased, physical injury (cf. immobility), heavy work load and night shifts causing sleep deprivation.<br>**Main difficulties:**<br>History of problematic family situation caused by severe alcohol dependency father. Sexual abuse in childhood resulting in lack of trust in men and sex addiction. Extremely depending on others.<br>**Diagnoses (DSM-IV):**<br>Depressive disorder; Relational problems; Obsessive compulsive PD	**Start**<br>**Post**<br>**1yFU**<br>**5yFU**	101<br>36<br>34<br>80
**Female, 35-40**	**Reason for consulting therapy centre:**<br>Suffering from CVS since adolescence. Lived at home all her life, not able to take care of herself resulting in isolation; violent conflicts with parents.<br>**Main difficulties:**<br>CVS: severe level of inability, dependence and isolation. Perfectionism, inability in dealing with emotions.<br>Unresolved past (e.g., victim of sexual harassment in childhood).<br>**Diagnoses (DSM-IV):**<br>Somatization disorder; PD NOS	**Start**<br>**Post**<br>**1yFU**<br>**5yFU**	83<br><br>56<br>92
**Male, 45-50**	**Reason for consulting therapy centre:**<br>"A crash", first physically (diagnosed with CVS), then mentally (depression). Verbal aggression towards wife and children (lead to seeking help). No experience with mental health care before.<br>**Main difficulties:**<br>History of violence in childhood (aggressive father). Perfectionism, inability in dealing with emotions (e.g., repressing anger and feelings in general), extreme fear of failure.<br>**Diagnoses (DSM-IV):**<br>Depressive disorder; Somatization disorder; Relational problems	**Start**<br>**Post**<br>**1yFU**<br>**5yFU**	108<br><br>54<br>48

**Male, 50-55**	**Reason for consulting therapy centre:**<br>Feelings of hopelessness and desperation. Outpatient care was experienced as insufficient.<br>**Main difficulties:**<br>A general feeling "not to be alive": existential crisis, experiencing life as meaningless. High demands and standards, not able to show vulnerability. History of "feeling different": as a child in his own family, later on in relation to friends and at work; history of rejection and exclusion. Suffering from fundamental loneliness.<br>**Diagnoses (DSM-IV):**<br>Depressive disorder (dysthymia); PD NOS	**Start**<br>**Post**<br>**1yFU**<br>**5yFU**	60<br>45<br>86<br>75
**Female, 55-60**	**Reason for consulting therapy centre:**<br>Feeling "stuck". Ambulant therapy sessions and short hospitalization in psychiatry were experienced as insufficient.<br>**Main difficulties:**<br>In general: difficult to live with self, interpersonal relations characterized by conflicts; difficulties in finding connection with others (ambivalence; distrust). Suffering from fundamental loneliness.<br>**Diagnoses (DSM-IV):**<br>Somatization disorder; Adjustment disorder; PD NOS	**Start**<br>**Post**<br>**1yFU**<br>**5yFU**	102<br>81<br>86<br>89
**Female, 35-40**	**Reason for consulting therapy centre:**<br>"Non-work-related burnout" (cf. extreme levels of stress and anxiety). Lived on "high speed" until "heavy crash" (e.g., social life, household became too much). Inpatient treatment necessary for being away from context.<br>**Main difficulties:**<br>History of psychological terror in family (aggressive father). Feelings of anxiety, high distress, easily overwhelmed. Low self-esteem and high need to please others.<br>**Diagnoses (DSM-IV):**<br>Anxiety disorder; PD NOS	**Start**<br>**Post**<br>**1yFU**<br>**5yFU**	68<br>52<br>61<br>71
**Female, 35-40**	**Reason for consulting therapy centre:**<br>Escalating relational problems; accumulating difficulties at work. Inpatient treatment necessary for being away from context.<br>**Main difficulties:**<br>Symbiotic relationship with mother (whom she describes as controlling and unable to give love). Therefore, not been able to develop own identity and personality apart from mother, leading to difficulties in other relationships (cf. highly depending on others).<br>**Diagnoses (DSM-IV):**<br>Adjustment disorder; PD NOS	**Start**<br>**Post**<br>**1yFU**<br>**5yFU**	91<br>61<br>73<br>67
**Female, 45-50**	**Reason for consulting therapy centre:**<br>Describes an overall feeling of "exhaustion" following family conflicts and marital problems. Break-up with life partner resulted in the loss of her stepson (and an important meaning in her life). Inpatient treatment necessary for being away from context.<br>**Main difficulties:**<br>Emotional vulnerability, no barriers to protect herself from influence and demands coming from others (extreme caring; over-empathic). Feeling different from others (and excluded from society).<br>**Diagnoses (DSM-IV):**<br>Depressive disorder; PD NOS	**Start**<br>**Post**<br>**1yFU**<br>**5yFU**	93<br><br>58<br>93
**Female, 45-50**	**Reason for consulting therapy centre:**<br>Severe depression after suicide attempt of husband and heavy care for children and household. Escalating relational problems (describes husband as manipulative and abusive); referred to inpatient therapy by relational therapist.<br>**Main difficulties:**<br>History of incest (childhood) and marital violence (psychological and physical).<br>**Diagnoses (DSM-IV):**<br>Depressive disorder; PTSD; Relational problems; PD NOS	**Start**<br>**Post**<br>**1yFU**<br>**5yFU**	94<br>77<br>80<br>67

**Female, 35-40**	**Reason for consulting therapy centre:**<br>Escalating problems in social and professional context (i.e., severe bullying and ending relationship), leading to increasing difficulties: inability to go to work, isolation from friends, not able to do household, to take care of self. Referred to inpatient treatment by psychiatrist.<br>**Main difficulties:**<br>Severe bullying in youth; physical violence in family. Mother with severe alcohol dependency; rejected by father and grandparents. Describes a "fear of men" and fear of rejection. Alcohol abuse (self).<br>**Diagnoses (DSM-IV):**<br>Depressive disorder; PD NOS	**Start** 142<br>**Post** 104<br>**1yFU** 124<br>**5yFU** 112
**Female, 60-65**	**Reason for consulting therapy centre:**<br>Combination of occurrences in private life and work (high perfectionism, "work" was as a life mission; burnout). Inpatient treatment necessary for being away from context.<br>**Main difficulties:**<br>Struggles with the existential question on the meaning of life without children.<br>**Diagnoses (DSM-IV):**<br>Somatization disorder; Obsessive compulsive PD	**Start** 88<br>**Post** 52<br>**1yFU** 53<br>**5yFU** 73
**Female, 55-60**	**Reason for consulting therapy centre:**<br>Inpatient treatment after two years of sick leave due to burnout. Inpatient treatment necessary for being away from context (high demanding: husband and mother require daily mental and practical care/help). Feeling of despair, not being heard by previous therapist (treatment insufficient).<br>**Main difficulties:**<br>Extreme caring for others; low self-esteem; fundamental feeling of being different and abnormal.<br>**Diagnoses (DSM-IV):**<br>Depressive disorder; Obsessive compulsive PD	**Start** 100<br>**Post** 49<br>**1yFU** 56<br>**5yFU** 68
**Female, 45-50**	**Reason for consulting therapy centre:**<br>Many depressive episodes were treated outpatient before hospitalization. Suicidal thoughts lead to psychiatric hospitalization prior to inpatient therapy.<br>**Main difficulties:**<br>Problematic relationship with parents (psychological neglect); constant feelings of guilt; extreme caring for others.<br>**Diagnoses (DSM-IV):**<br>Depressive disorder; Somatization disorder; Avoidant PD; Dependent PD	**Start** 83<br>**Post** 40<br>**1yFU** 52<br>**5yFU** 66
**Female, 55-60**	**Reason for consulting therapy centre:**<br>"A breakdown" due to pressure at work. Depression, feeling of having "no way out".<br>**Main difficulties:**<br>Got separated from family at birth, reunited at age three. Has felt excluded by parents and siblings ever since. Suffered from dyslexia, which was only discovered lately. Low self-esteem as a consequence of abundant negative feedback on her cognitive abilities. Different person to the outside world. Difficulties in social situations (e.g., she cannot enter a public place alone or first).<br>**Diagnoses (DSM-IV):**<br>Depressive disorder; Avoidant PD; Dependent PD	**Start** 113<br>**Post** 115<br>**1yFU** 90<br>**5yFU** 93
**Female, 45-50**	**Reason for consulting therapy centre:**<br>Severe depression following escalating situation at home. Unable to work due to fibromyalgia (cf. isolation). Increasing feelings of loneliness and anxiety when alone at home and feelings of guilt towards family members (cf. not able to contribute to household). Inpatient treatment as a means of giving time, space and rest to all family members.<br>**Main difficulties:**<br>History of neglect as an infant (describes mother as cold, careless). Previous psychiatric treatment (as a teenager; two years of hospitalization) for anorexia nervosa.<br>**Diagnoses (DSM-IV):**<br>Depressive disorder (dysthymia); Relational problems; Dependent PD; Avoidant PD	**Start** 91<br>**Post** 87<br>**1yFU** 69<br>**5yFU** 66
**Female, 55-60**	**Reason for consulting therapy centre:**<br>General feeling of "not doing well" (regarding self, at work and in relationship). Referred to inpatient treatment by psychiatrist.<br>**Main difficulties:**<br>Child deceased due to a drug overdose; she feels guilty for his death. Violent marital relationship with ex-	**Start** 101<br>**Post** 73

	husband (psychological and physical aggression). Heavy depression and suicide attempt during that period. Autism (diagnosed after inpatient treatment) explains difficulties in relationships.<br>**Diagnoses (DSM-IV):**<br>Adjustment disorder; PD NOS	**1yFU**<br>**5yFU**	66<br>95
**Female, 60-65**	**Reason for consulting therapy centre:**<br>General anxiety and difficulties in relationship (e.g., dominant husband).<br>**Main difficulties:**<br>Father deceased when she was a child (she was not informed nor involved in the sickness process). Describes her mother as highly dominant (cf. breaking her down, inducing feelings of guilt). Anxiety (all her life, in relation to family, friends, partners: afraid not to be good enough, to do something wrong).<br>**Diagnoses (DSM-IV):**<br>Depressive disorder; PD NOS	**Start**<br>**Post**<br>**1yFU**<br>**5yFU**	78<br>74<br>68<br>82
**Female, 30-35**	**Reason for consulting therapy centre**:<br>After years of psychiatric treatment and individual therapy, referred to inpatient psychotherapy to address unresolved family issues and sexual abuse in childhood.<br>**Main difficulties:**<br>History of sexual abuse (childhood). Several hospitalizations in psychiatric care (three years in total) for the treatment of anorexia nervosa. Extreme fear of failure and perfectionism leading to depression and eating disorder (as a teenager). Difficult relationship with family following abuse and psychological difficulties. Negative body image.<br>**Diagnoses (DSM-IV):**<br>Adjustment disorder; PTSD; Borderline PD	**Start**<br>**Post**<br>**1yFU**<br>**5yFU**	144<br>60<br>107<br>80
**Female, 35-40**	**Reason for consulting therapy centre:**<br>Escalation of problems: accident (long revalidation); heavy job (working at night, no social life, isolation, sleep deprivation); sexual harassment (afraid to go out at night); unhealthy meals. Looked for help after aggressive outbursts at work. Reacted heavily (physically) on medication and was referred to inpatient treatment.<br>**Main difficulties:**<br>Describes family as harsh: speaking about difficulties and emotions is not done. Highly self-demanding (e.g., does not rest when ill). General distrust in others.<br>**Diagnoses (DSM-IV):**<br>Depressive disorder	**Start**<br>**Post**<br>**1yFU**<br>**5yFU**	94<br><br>44<br>48
**Female, 40-45**	**Reason for consulting therapy centre:**<br>"A crash" due to heavy care for children, all diagnosed with autism. Depression.<br>**Main difficulties:**<br>Difficulties due to (own) autism (diagnosed only years after inpatient treatment). Perfectionism; being easily overwhelmed. Difficulties in communicating feelings. Not easy to let others in (emotionally).<br>**Diagnoses (DSM-IV):**<br>Adjustment disorder; PD NOS	**Start**<br>**Post**<br>**1yFU**<br>**5yFU**	73<br><br>102<br>116

Note *n* = 22. Information based on patients' own description of complaints, life history and reason for consulting the therapy centre as reported during the follow-up interview five to six years after ending therapy. Note that these brief clinical descriptions have the intention to provide more background information on patients (history, complaints other than symptomatic difficulties), but cannot be considered a thorough and complete anamnesis. Outcome scores: missing values were left blank. DSM-IV diagnoses as assessed during the intake period, prior to inpatient treatment; information was provided by the centre's psychiatrist.
